# Dynamic Alterations in Spontaneous Neural Activity in Multiple Brain Networks in Subacute Stroke Patients: A Resting-State fMRI Study

**DOI:** 10.3389/fnins.2018.00994

**Published:** 2019-01-07

**Authors:** Jing Chen, Dalong Sun, Yonghui Shi, Wei Jin, Yanbin Wang, Qian Xi, Chuancheng Ren

**Affiliations:** ^1^Department of Neurology, Shanghai Fifth People’s Hospital, Fudan University, Shanghai, China; ^2^Division of Gastroenterology, Department of Internal Medicine, Zhongshan Hospital, Fudan University, Shanghai, China; ^3^Department of Radiology, Shanghai East Hospital, Tongji University, Shanghai, China; ^4^Department of Neurology, Shanghai East Hospital, Tongji University, Shanghai, China

**Keywords:** stroke, dynamic intrinsic brain activity, resting-state fMRI, amplitude of low-frequency fluctuations, regional homogeneity

## Abstract

**Objective:** To examine whether subacute stroke patients would exhibit abnormal dynamic characteristics of brain activity relative to healthy controls (HC) and to investigate whether the altered dynamic regional indexes were associated with clinical behavior in stroke patients.

**Methods:** The dynamic amplitude of low-frequency fluctuations (dALFF) and dynamic regional homogeneity (dReHo) in 42 subacute stroke patients and 55 healthy controls were compared. Correlation analyses between dALFF and dReHo in regions showing significant intergroup differences and clinical scores (i.e., the National Institutes of Health Stroke Scale, Fugl-Meyer assessment and lesion volume size) were conducted in stroke patients. Receiver operating characteristic (ROC) curve analysis was used to determine the potential value of altered dynamic regional indexes to identify stroke patients.

**Results:** Significantly dALFF in the bilateral cerebellum posterior lobe (CPL), ipsilesional superior parietal lobe, ipsilesional inferior temporal gyrus (ITG), the midline supplementary motor area (SMA), ipsilesional putamen and lentiform nucleus were detected in stroke patients compared to HC. Relative to the HC group, the stroke patients showed significant differences in dReHo in the contralesional rectal gyrus, contralesional ITG, contralesional pons, ipsilesional middle frontal gyrus (MFG). Significant correlations between dALFF variability in midline SMA and Fugl-Meyer assessment (FMA) scores or between dReHo variability in the ipsilesional MFG and FMA scores were detected in stroke patients. Furthermore, the ROC curve revealed that dynamic ALFF at SMA and ReHo at ipsilesional MFG might have the potential to distinguish stroke patients.

**Conclusion:** The pattern of intrinsic brain activity variability is altered in stroke patients compared with HC, and dynamic ALFF/ReHo might be potential tools to assess stroke patients’ motor function.

## Introduction

Stroke is the most common cause leading to varying degrees of neurological dysfunction with a very high likelihood of long-term disability (Liu et al., 2011; Yang et al., 2013). Movement disorders are the major common conditions of stroke-induced disability, and motor functional recovery remains highly variable. Although the exact mechanism of motor deficits and motor recovery are still under investigation, recent advances in neuroimaging have expanded our understanding. Resting-state functional magnetic resonance imaging (fMRI), which is operationally defined as task-independent spatiotemporal correlations within functionally related regions of the brain (Biswal et al., 1995), has been extensively used to delineate neural function abnormalities in stroke patients.

Resting-state fMRI measures spontaneous brain activity in low-frequency fluctuations which can be reflected by the blood oxygen level dependent (BOLD) signal. An increasingly large body of resting-state fMRI studies in stroke patients has focused on the characteristics of within-region or inter-region functional connectivity, such as connections within motor networks or between motor networks and non-motor networks (Wang et al., 2010; Grefkes and Fink, 2014; Wu et al., 2015). However, few studies have examined regional brain activities in patients with stroke. Neural regional properties are crucial for a better understanding of the neurophysiological and neuropathological conditions, such as regional abnormal energy consumption suggesting excessive or decreased resting metabolic rates (Raichle, 2006; Fox and Raichle, 2007). Currently, one of the methods to measure regional properties of the BOLD signal is the amplitude of low-frequency fluctuations (ALFF), which measures the signal strength in low-frequency oscillations of spontaneous neural activity (Zang et al., 2007). The ALFF is correlated with field potential activity in local brain regions (Logothetis et al., 2001), and the amplitude of oscillations can be applied as an index to examine alterations in neural function (Mohamed et al., 2004). Another approach is regional homogeneity (ReHo), which reflects the statistical similarity of local neural activity among spatially adjacent regions (Zang et al., 2004). These two approaches have been widely adopted for evaluating local neural function in neurologic disorders and neuropsychiatric diseases (Qiu et al., 2011; Li et al., 2012; Liu et al., 2012, 2013).

It has been reported that ALFF or ReHo were altered under resting conditions in stroke patients with movement disorders (Skidmore et al., 2013; Tsai et al., 2014) and that the ALFF value or ReHo value in certain brain regions were associated with the severity of motor deficits (Liu et al., 2015; Zhu et al., 2015). However, the aforementioned investigations of regional brain activities assumed that the BOLD signal is stationary during the entire fMRI scan, ignoring the characteristics of dynamic changes of brain spontaneous activity over time. Indeed, evidence has accumulated that brain responds to internal or external stimuli by dynamic integration or adjustment over multiple time scales (Abrams et al., 2013; Yin et al., 2013). Fortunately, the dynamic nature of brain activity may be detected by task manipulations using methods such as electroencephalography and can also be informed by the lower temporal resolution of resting-state fMRI (Calhoun et al., 2008). In recent years, sliding window approaches to functional connectivity have effectively examined abnormal brain function in stroke (Duncan and Small, 2017). Nevertheless, it is not enough to merely focus on time-varying dynamic functional connectivity, since evidence from neuroimaging techniques of high spatiotemporal resolution has verified that local brain activity itself exists with substantial fluctuations (Liao et al., 2015; Fu et al., 2017), and until now, no study explored the dynamic characteristics of local brain activity indexes in stroke patients. These dynamic local approaches are expected to explore the variability of the oscillation amplitudes and regional synchronization of spontaneous brain activity and to advance our understanding of brain function by identifying specific pathophysiological function signatures and our ability to decipher the neural underpinnings of normal or abnormal human behaviors.

Hence, the present study applied resting-state fMRI to investigate whether subacute stroke patients would exhibit abnormal dynamic characteristics of spontaneous brain activity by calculating regional indexes, ALFF and ReHo, compared with healthy controls (HC). Furthermore, another goal was to explore whether the altered dynamic ALFF (dALFF) and dynamic ReHo (dReHo) were correlated with the clinical behavior of the stroke patients. In the current study, we included subacute stroke patients for two reasons. First, the condition of stroke patients at the subacute stage is relatively stable than acute stroke patients, and the patients’ compliance is relatively high, facilitating the smooth progress of the current study. Second, we enrolled stroke patients within 1–3 weeks after symptom onset, and this period is well within the recovery window. A period of dramatic changes in functional and structural reorganization may provide more information of spontaneous neural activity. We hypothesized that variability of regional brain activity was altered in patients with stroke-induced motor deficits compared with HC and that dynamic regional indexes in certain regions detected to be associated with the Fugl-Meyer assessment (FMA) scores could provide more information for evaluating of motor function in stroke patients.

## Materials and Methods

### Subjects

This study was part of an integrated stroke and rehabilitation project at Shanghai 5th People’s Hospital affiliated with Fudan University and was approved by the local ethical committee of Shanghai 5th People’s Hospital affiliated with Fudan University. Written informed consent was obtained from all subjects before participating according to the Declaration of Helsinki. A total of 45 subacute stroke patients were recruited. Additionally, 55 HC, who were right-handed and matched for age, gender and education, were recruited from the local communities. The inclusion criteria for stroke patients were as follows: (1) they were aged 40–80 years; (2) it was a first-onset stroke with a single lesion in right-side subcortical regions as verified by diffusion-weighted imaging (DWI); (3) they were examined within 1–3 weeks after stroke symptom onset; (4) they were clinical evidence of a motor deficit based on neurological examination; and (5) they were right-handed before the stroke. The exclusion criteria for both stroke patients and HC were the presence of any of the following: (1) other brain abnormalities, or clinically significant or unstable medical diseases; (2) unconsciousness, cognitive impairment, or cooperation difficulties; (3) patients with use of medications that could affect motor examination, such as antipsychotics and antiepileptics; (4) patients with cerebellar lesions; and (5) contraindications for MRI scanning. For all stroke patients, the right hemisphere corresponded to the ipsilesional hemisphere. The National Institutes of Health Stroke Scale (NIHSS) and Mini-Mental State Examination (MMSE) were used to evaluate neurological function impairment and cognitive conditions. FMA for upper and lower extremities was applied to evaluate the degree of motor deficit. These clinical behavior scores were collected on the same day as fMRI data acquisition.

### Data Acquisition

All resting-state fMRI data were acquired using a Philips Achieva 3.0 T MR scanner (Philips Medical Systems, Best, Netherlands). Tight but comfortable foam pads and earplugs were used to reduce head motion and scanner noise. Resting-state fMRI was collected using an echo-planar (EPI) sequence with the following scan parameters: repetition time (TR) = 2000 ms; repetition echo time (TE) = 30 ms; flip angle (FA) = 90°; field of view (FOV) = 220 mm × 220 mm; voxel size = 3 mm × 3 mm × 3 mm; matrix = 64 × 64; slice thickness = 3 mm; gap = 1 mm; interleaved transversal slices = 38; and number of volumes = 180. High-resolution sagittal T1-weighted images were acquired using a 3D magnetization prepared rapid gradient echo (MPRAGE) sequence: TR = 8.0 ms; TE = 3.7 ms; FA = 12°; FOV = 256 mm × 256 mm; voxel size = 1 mm × 1 mm × 1 mm; matrix = 256 × 256; slice thickness = 1 mm; and slices = 180. During scanning, all participants were instructed to remain awake, keep their eyes closed, and stay motionless without thinking of anything in particular.

**FIGURE 1:**
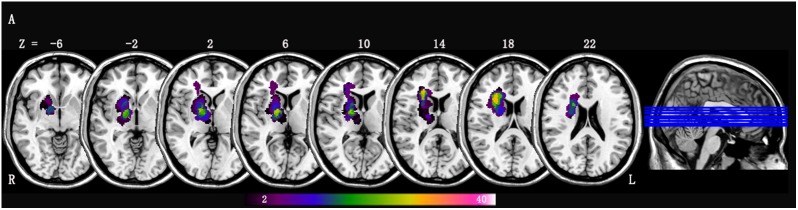
Lesion overlap map across stroke patients with right-sided lesions (*n* = 42); Lesion maps were normalized to an MNI reference brain. The color bar indicates the percentage of lesion overlap.

The lesion location of each patient was determined by an experienced neuroradiologist on T1-weighted MRI images. We manually outlined the lesion profiles on T1-weighted MRI images slice by slice using the software MRIcron^1^ and generated a lesion mask for each patient. After spatial normalization to Montreal Neurological Institute (MNI) space, all the patients’ lesion masks overlapped. We then averaged the individual lesion masks and overlaid them with a template to create the lesion overlap map shown in Figure 1.

### Preprocessing of Resting-State fMRI Data

The preprocessing of resting-state fMRI data was performed using the Data Processing Assistant for Resting-State fMRI (DPARSF) version 4.0^2^. The first 10 volumes of each participant were deleted to allow the signal to reach equilibrium and the subjects to adapt to the environment. The remaining 170 volumes were corrected for acquisition time delay between slices. Realignment was conducted to correct head motion. The participants with head motion of >2.0 mm in maximum displacement or >2.0° rotation in angular motion were excluded from the study. The mean framewise displacement (FD) was computed by averaging the FD of each subject across the time points, and no significant differences were found between stroke patients and HC (*p* = 0.235). In addition, each subject’s mean FD was included in all group-level analyses as a covariate to further control the head move effect. Subsequently, the structural image was coregistered to the mean functional image after the motion correction, and the transformed structural image was segmented into gray matter, white matter, and cerebrospinal fluid. Then, the segmented images were normalized to MNI space using Diffeomorphic Anatomical Registration Through Exponentiated Lie algebra (DARTEL) algorithm (Ashburner, 2007). Next, the motion-corrected functional volumes were normalized to the MNI space using the normalization parameters for their respective structure images and resampled into a voxel size of 3 mm × 3 mm × 3 mm. Nuisance covariates (24 head motion parameters, cerebrospinal fluid signal, white matter signal and linear trend) were regressed out. Given that it is still a controversy of removing the global signal (Murphy et al., 2009); we did not regress out the global signal. For the ReHo calculation, an additional processing step was that the regressed functional images were temporally bandpass filtered (0.01–0.08 Hz) to reduce low frequency drift and high-frequency noise.

### dALFF and dReHo Analysis

Dynamic regional metrics analysis was performed using Temporal Dynamic Analysis (TDA) toolkits based on DPABI (Yan et al., 2017). Sliding window-based analysis, which is sensitive in detecting time-dependent variations (Hindriks et al., 2016; Liu F. et al., 2017; Yip et al., 2017), was applied to examine the dALFF or dReHo variability over the whole brain. In the sliding window analysis, a temporal window of certain size and shape is chosen, and ALFF and ReHo within that window are calculated. Ideally, the window size should be small enough to detect potentially transient signals, and yet large enough to analyze the lowest frequencies of interest in the signals (Sakoglu et al., 2010). Previous work of sliding window connectivity have applied a sliding window length of as small as 10 s (Thompson et al., 2013) and as long 180 s (Gonzalez-Castillo et al., 2015). In this work, a moderate-length sliding window of 32 TR (64 s) and a shifting step size of one TR (2 s) were used to simultaneously maximize statistical power within the window and also maximize statistical power for cross-level analyses (Allen et al., 2014). The remaining 170 time points after removing the first 10 time points for each individual were segmented into 139 windows in total. In each sliding window, ALFF and ReHo were calculated. For ALFF, the time series was first converted to the frequency domain using a fast Fourier transform, and then the ALFF value of a given voxel was obtained by calculating and summing the square root of the power spectrum between 0.01 and 0.08 Hz. For ReHo, the Kendall’s coefficient of concordance (KCC) of the time course of every 27 nearest neighboring voxels was calculated (Zang et al., 2004). The standard deviation (SD) of ALFF values and ReHo values at each voxel across 139 windows was calculated to assess the variability of ALFF and ReHo. To reduce the global effects of variability across subjects, the dALFF and dReHo of each voxel were divided by the global mean dALFF and dReHo values within a gray matter mask, respectively. Finally, the mean normalized dALFF and dReHo maps were spatially smoothed with an isotropic Gaussian kernel of 4 mm full-width-at-half-maximum (FWHM).

### Statistical Analysis

A general linear model (GLM) was used in a voxel-wise manner to compare group differences between the stroke group and HC group in dALFF and dReHo with age, gender, educational level, MMSE and mean FD as covariates. Multiple comparisons were corrected using a voxel-level familywise error rate (FWE) method with corrected *p* < 0.05 (cluster size ≥50 voxels).

The Shapiro–Wilk statistic was first used to test for normality, and then group comparisons of clinical measures were performed using two-sample *t*-tests for continuous data and Pearson’s chi-squared test for categorical data. Partial correlation analyses were conducted in stroke patients between the clinical measures (NIHSS scores, FMA scores and lesion volume size) and the mean dALFF/dReHo value of each cluster showing significant group differences between stroke group and HC group. The age, gender, educational level, MMSE, mean FD, illness duration and intravenous thrombolysis (IVT) were also considered as covariates. As the correlation analyses were exploratory in nature, the significance levels were set at uncorrected *p* < 0.05. Furthermore, receiver operating characteristic (ROC) curve analysis was performed to examine the potential value of altered dynamic ALFF or ReHo values in clusters showing significant correlations with clinical behaviors in stroke patients. The optimal cut-off between sensitivity and specificity was determined by maximizing the Youden’s index J (J = sensitivity + specificity - 1). A two-tailed *p*-value of 0.05 was considered statistically significant for the analyses conducted with SPSS version 19.0 statistical software (IBM Corporation, Armonk, NY, United States).

## Results

### Clinical Data

Data obtained from three stroke patients were excluded because of excessive head motion during scanning. Demographic and clinical characteristics of 42 patients with right hemisphere stroke (21 men; mean age, 57.86 ± 11.17 years) and 55 HC (29 men; mean age, 56.73 ± 10.21 years) are listed in Table 1. No significant differences in were found (*p* > 0.05) in gender, age, education level, high risk factors (hypertension, diabetes, hyperlipidemia and atrial fibrillation) and MMSE scores between the stroke group and HC group. The mean lesion volume of stroke patients was 6.10 ± 8.55 cc^3^. Among them, four patients received IVT therapy. Of the 42 patients, 15 had corona radiate lesions, 15 had internal capsule lesions, 10 had basal ganglia lesions, and two had thalamus lesions. The lesion overlaps of stroke patients are shown in Figure 1.

**Table 1:** Demographic and clinical data.

	Stroke patients (*n* = 42)	Healthy control (*n* = 55)	*t*/χ^2^	*p*
Gender, male	21 (50.0)	29 (52.7)	0.071	0.790
Age, years	57.86 ± 11.17	56.73 ± 10.21	0.518	0.605
Educational level, years	10.74 ± 3.39	11.00 ± 3.82	-0.351	0.727
**High risk factor**				
Hypertension	25 (59.5)	29 (52.7)	0.446	0.504
Diabetes	11 (26.2)	12 (21.8)	0.252	0.616
Hyperlipidemia	12 (28.6)	16 (29.1)	0.003	0.955
Atrial fibrillation	2 (2.1)	1(1.8)	0.689	0.407
MMSE	28.07 ± 1.28	28.15 ± 1.27	-0.284	0.777
Illness duration, days	14.29 ± 2.14	-		
**Stroke type**				
Ischemia	40 (95.2)	-		
Hemorrhage	2 (4.8)	-		
**Location of lesion**				
Coronal radiate	15 (35.7)	-		
Internal capsule	15 (35.7)	-		
Basel ganglia	10 (23.8)	-		
Thalamus	2 (4.8)	-		
Lesion volume, cc^3^	6.10 ± 8.55	-		
NIHSS	5.86 ± 4.14	-		
FMA-total	76.45 ± 14.12	-		
IVT use	4 (9.5)	-		


*Data represent *n* (%) or mean ± SD. MMSE, the Mini-Mental State Examination; NIHSS, the National Institutes of Health Stroke Scale; FMA-total, Fugl-Meyer assessment for upper and lower extremities; IVT, intravenous thrombolysis.*

### Differences in Dynamic ALFF and Dynamic ReHo

**FIGURE 2:**
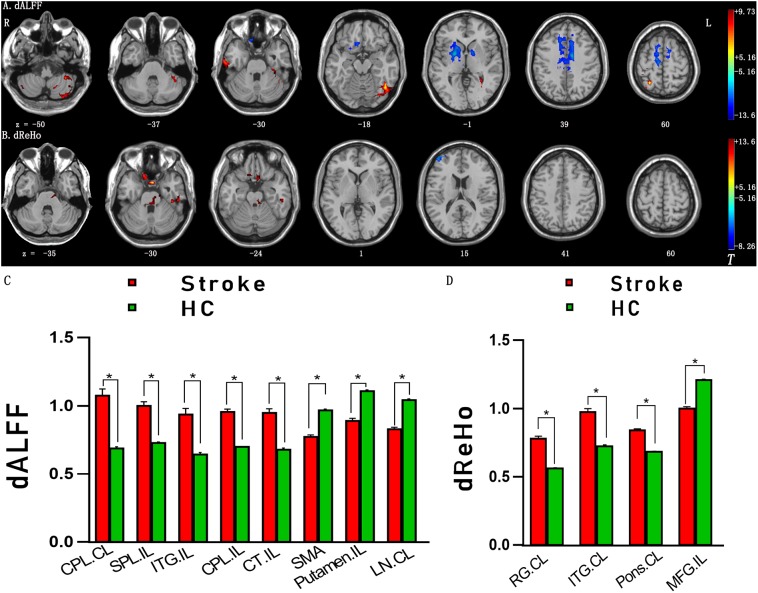
**(A)** Brain regions with significant intergroup differences in dALFF between the stroke group and HC group. **(B)** Brain regions with significant intergroup differences in dReHo between the stroke group and HC group. **(C)** The histogram indicates mean values and standard error of dALFF variability between the stroke group and HC group. **(D)** The histogram indicates mean value and standard error of dReHo variability between the stroke group and HC group. Familywise error rate corrected (*p* < 0.05, cluster size ≥ 50 voxels). The color bar indicates the *T* value. ^∗^*p* < 0.05. HC, healthy controls; dALFF, dynamic amplitude of low-frequency fluctuation; dReHo, dynamical regional homogeneity; CPL, cerebellum posterior lobe; SPL, superior parietal lobe; ITG, inferior temporal gyrus; CT, cerebellum tonsil; SMA, supplementary motor area; LN, lentiform nucleus; RG, rectal gyrus; MFG, middle frontal gyrus; IL, ipsilesional; CL, contralesional.

The significant differences in dALFF and dReHo between the stroke group and HC group are shown in Figures 2A,B, respectively. Compared with HC, significantly increased dALFF variability in the contralesional cerebellum posterior lobe (CPL), ipsilesional superior parietal lobe, ipsilesional inferior temporal gyrus (ITG), ipsilesional CPL and cerebellum tonsil and decreased dALFF variability in the midline supplementary motor area (SMA), ipsilesional putamen and lentiform nucleus were detected in stroke patients (Table 2 and Figures 2A,C). Relative to the dReHo variability of subjects in the HC group, these stroke patients showed a significant increase in contralesional rectal gyrus, contralesional ITG, contralesional pons and a significant decrease in ipsilesional middle frontal gyrus (MFG) (Table 3 and Figures 2B,D).

**Table 2:** Brain regions with significant differences in dynamic ALFF between groups.

		Peak MNI coordinates				
				
Group comparisons	Brain regions/BA	X	Y	Z	Cluster size (voxels)	Peak *t* values
Stroke patients > HC	Cerebellum posterior lobe, CL	-15	-69	-39	1175	9.73
	Superior parietal lobe, IL/4	27	-57	60	179	9.32
	Inferior temporal gyrus, IL/20	39	-57	-18	298	9.27
	Cerebellum posterior lobe, IL	39	-87	-39	53	8.33
	Cerebellum tonsil, IL	48	-63	-54	50	6.57
Stroke patients < HC	Supplementary motor area, 6	12	-12	57	1670	13.6
	Putamen, IL	24	0	0	814	12.18
	Lentiform nucleus, CL	-18	-6	18	298	11.01


*ALFF, amplitude of low-frequency fluctuation; HC, healthy controls; BA, Brodmann’s area; MNI, Montreal Neurological Institute; IL, ipsilesional; CL, contralesional.*

**Table 3:** Brain regions with significant differences in dynamic ReHo between groups.

		Peak MNI coordinates				
				
Group comparisons	Brain regions/BA	X	Y	Z	Cluster size (voxels)	Peak *t* values
Stroke patients > HC	Rectal gyrus, CL/11	-3	6	-30	105	13.16
	Inferior temporal gyrus, CL/20	-42	-30	-30	58	8.85
	Pons, CL	-12	-21	-33	95	7.86
Stroke patients < HC	Middle frontal gyrus, IL/9	45	54	18	52	7.32


*ReHo, regional homogeneity; HC, healthy controls; BA, Brodmann’s area; MNI, Montreal Neurological Institute; IL, ipsilesional; CL, contralesional.*

### Correlational Analysis

**FIGURE 3:**
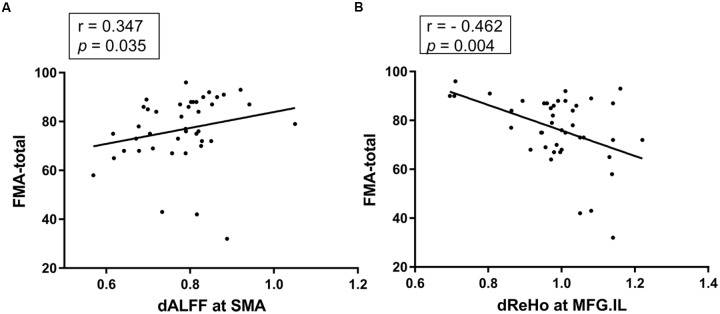
**(A)** The dALFF variability in the SMA was positively correlated with the FMA scores of the stroke patients (*r* = 0.347, *p* = 0.035, uncorrected). **(B)** The dReHo variability in the ipsilesional MFG was negatively correlated with the FMA scores of the stroke patients (*r* = –0.462, *p* = 0.004, uncorrected). dALFF, dynamic amplitude of low-frequency fluctuation; dReHo, dynamic regional homogeneity; FMA-total, Fugl-Meyer assessment for upper and lower extremities; SMA, supplementary motor area; MFG, middle frontal gyrus; IL, ipsilesional.

A significant positive correlation was detected between the FMA scores and dALFF variability in the SMA (*r* = 0.347, *p* = 0.035, uncorrected; Figure 3A), a significant negative correlation between the FMA scores and dReHo variability in the midline ipsilesional MFG was found in stroke patients (*r* = -0.462, *p* = 0.004, uncorrected; Figure 3B). No other significant correlations between NIHSS, size of lesion volume and dynamic regional indexes were observed in the stroke group.

### ROC Analysis

**FIGURE 4:**
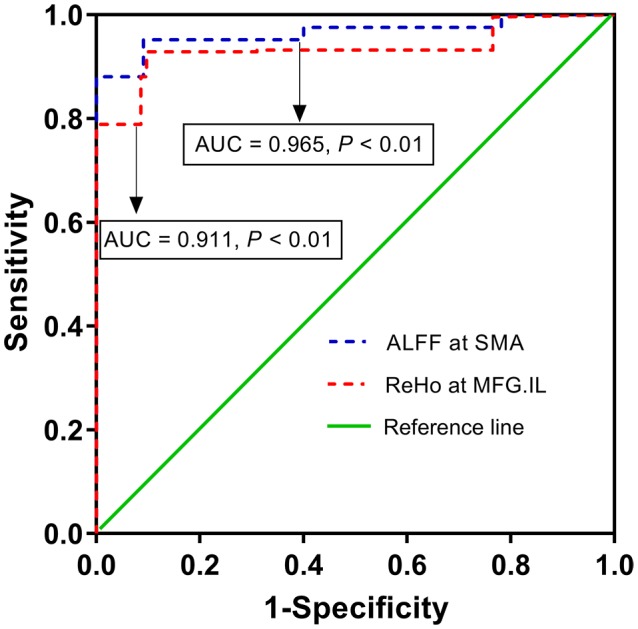
The diagnostic performance of altered dynamic ALFF in SMA and dynamic ReHo in ipsilesional MFG in distinguishing stroke patients from healthy subjects. ALFF, amplitude of low-frequency fluctuation; ReHo, regional homogeneity; SMA, supplementary motor area; MFG, middle frontal gyrus; IL, ipsilesional; AUC, area under curve.

As shown above, significant correlations were detected between the FMA scores and dynamic ALFF variability in SMA or dynamic ReHo variability in ipsilesional MFG, which proposed that the dynamic ALFF/ReHo in these regions might be utilized to differentiate the stroke patients from healthy persons. To verify this possibility, mean dALFF/dReHo values in the SMA or ipsilesional MFG were extracted. Then, ROC analysis was performed to investigate this possibility. The results demonstrated that the area under the curves (AUC) of SMA and ipsilesional MFG were 0.965 and 0.911, respectively (Figure 4), which suggested that dynamic ALFF in SMA and ReHo values in the ipsilesional MFG might have the potential to distinguish the stroke patients from healthy subjects. Further diagnostic analysis showed that the sensitivity and specificity were relatively high (Table 4).

**Table 4:** ROC analysis for differentiating stroke patients from healthy person.

	MNI coordinates					
				
Brain regions	X	Y	Z	AUC	Maximal Youden’s index J	Sensitivity	Specificity
dALFF at SMA	12	-12	57	0.965	0.861^*^	95.2% (40/42)	90.9% (50/55)
dReHo at MFG.IL	45	54	18	0.911	0.832	92.9% (39/42)	90.3% (49/55)


*^∗^By this optimal cut-off point, the dynamic ALFF value of SMA could correctly classify 40 of 42 patients and 50 of 55 healthy subjects, resulted in a sensitivity of 95.2% and a specificity of 90.9.2%. The means of other optimal cut-off point was similar. MNI, Montreal Neurological Institute; AUC, areas under the curves; dALFF, dynamic amplitude of low-frequency fluctuation; dReHo, dynamic regional homogeneity; SMA, supplementary motor area; MFG, middle frontal gyrus; IL, ipsilesional.*

## Discussion

In the present study, dynamic regional brain activity between stroke patients with motor deficits and healthy controls was examined using resting-state fMRI. To the best of our knowledge, there is no prior study using a TDA approach to detect stroke-related brain activity changes in humans. Given that young adult stroke could be different both in causes and in outcomes (Edwards et al., 2018) and the role of educational level in recovery should not be ignored (Hillis and Tippett, 2014). Therefore, we used age and educational level as covariates in intergroup difference analyses and correlation analyses. Moreover, patients were enrolled in this current study within 1–3 weeks after stroke symptom onset, during which period imaging data and behavior performance were obtained. This period is well within the recovery window for stroke (Venketasubramanian et al., 2017). Additionally, medication, especially IVT therapy, can largely improve patients’ functional outcomes for hyperacute ischemic stroke (Ferrigno et al., 2018). Hence, we also considered patients’ illness duration and IVT use status as covariates in correlation analyses to reduce the confounding effects.

Differences in dALFF variability were observed between the stroke group and HC group in the contralesional CPL, ipsilesional superior parietal lobe, ipsilesional ITG, ipsilesional CPL, cerebellum tonsil, the midline SMA, ipsilesional putamen and lentiform nucleus, while differences of dReHo variability in contralesional rectal gyrus, contralesional ITG, contralesional pons and MFG distinguished stroke patients from HC. In addition, relationships were observed between the FMA scores and dynamic ALFF or ReHo variability in SMA or ipsilesional MFG in stroke patients. Further ROC analyses suggested that dynamic ALFF in SMA or ReHo in ipsilesional MFG had the potential to distinguish the patients of subacute stroke from healthy subjects.

Our findings were in line with previous studies that investigated stroke patients with movement disorders by functional connectivity, structural connectivity or regional metrics, such as ALFF or ReHo (Skidmore et al., 2013; Tsai et al., 2014; Zhu et al., 2015). The sensorimotor network (SMN), which is critical for voluntary movement, connects primary motor cortex function with SMA function (Damoiseaux et al., 2006; Chang et al., 2013). The SMA is a part of the primate cerebral cortex that contributes to the control of movement. Neurons in the SMA project directly to the spinal cord and may play a role in the direct control of movement (Nudo and Masterton, 1990). It has been reported that SMA plays roles in the postural stabilization of the body, the coordination of both sides of the body such as during bimanual action, the control of movements that are internally generated rather than triggered by sensory events, and the control of sequences of movements (Shima and Tanji, 1998; Cunnington et al., 2003; Zhang et al., 2012). Stroke-induced disturbance of intrinsic neural activity, which may be due to a complex cascade of events that are associated with structural reorganization and axonal sprouting as demonstrated by tract-tracing studies in animal models of stroke (Carmichael et al., 2001; Dancause et al., 2005), impedes brain network function of voluntary motion. Based on prior studies, we speculate that altered dALFF and dReHo variability in the SMN (e.g., in the superior parietal lobe and SMA) of stroke patients with motor deficits might occur as a compensatory mechanism and might be a significant factor in the reorganization and integration of resting-state functional networks at the subacute stage.

We observed decreased dReHo variability in the ipsilesional MFG, which belongs to the default mode network (DMN). This finding may indicate that stroke-related brain activity changes not only occurred in motor-related areas but also in non-motor regions. The DMN plays a pivotal role in “resting” brain activity, which is involved in sustaining attention, self-consciousness and exhibiting self-control (Andrews et al., 2011; Yuan et al., 2018). In stroke patients, alterations in brain activity of the DMN may be associated with advanced neural function of cognitive and emotional control (Liu F. et al., 2017). Evidence has shown that the DMN regulates consciousness, processes emotionally salient stimuli, and coordinates the interactions of cognitive function and emotional processing (Soddu et al., 2012; Zhang et al., 2017). In the current study, we demonstrated that the stroke group showed significantly decreased ReHo variability in the ipsilesional MFG, indicating that the synchronous neural activity was also disrupted in the ipsilesional MFG in stroke patients.

The increased regional brain activity in contralesional pons and bilateral cerebellum CPL, ipsilesional cerebellum tonsil may result from the dysfunction of the cerebro-ponto-cerebellar circuit and act as a compensatory response (Lu et al., 2011). The excessive ReHo variability of the prefrontal cortex (the rectal gyrus) may compensate for the deficits in motor function in stroke patients. However, this concept needs to be confirmed. Moreover, rectal gyrus is considered as a part of affective network (AN). Study has reported that abnormal regional neural activity was observed mainly in component of DMN, SMN, cerebellar lobes (CPL) and AN in stroke patients with emotional abnormality (Liu T. et al., 2017). Hence, we tend to speculate that the dysfunction of the volitional system may lead to the disorder of emotional system in stroke patients.

We also found decreased dALFF variability in the ipsilesional putamen and contralesional lentiform nucleus in stroke patients. Considered the entrance to the basal ganglia, the lentiform nucleus receives the input from cerebral cortex, and there was partial stroke lesion overlap observed in the present study and shown in Figure 1. The primary roles of these regions are to regulate movements at various stages, such as during motor preparation and motor execution. Meanwhile, they play important roles in motor learning, which can be considered a sensory feedback of frontal mediated goal parameters and posterior-mediated motor programs; there are anatomical links that exist between these motor-execution and frontal-parietal motor control systems (Ween, 2008). Previous human and non-human studies of stroke models have shown that decreased ALFF values were found in the core of stroke lesions (Liu et al., 2007; Skidmore et al., 2013). The mechanism may involve abrupt decreases of the blood flow resulting from cytotoxic swelling, calcium overload and membrane ion pump failure in core regions of stroke lesions (Badaut et al., 2011). Based on the previous studies and this present study, we infer that deceased or vanished oxygen consumption and intrinsic brain activity may contribute to less variation and low ALFF values.

In addition, we also detected excessive intrinsic brain activity variability reflected both in dALFF in the ipsilesional ITG and in dReHo in the contralesional ITG of the stroke patients. The ITG belongs to the higher levels of the ventral stream of visual processing and is related to the representation of complex object features. A previous study found that the visual network was activated in recovery from sensorimotor stroke, and limb movement critically relies on visual guidance (Archer et al., 2016). The mechanism underlying the abnormal brain activity of contralesional ITG is presently poorly understood.

The current study has several limitations. First, although we identified significant differences between the stroke patients and the HCs, the sample size of 42 stroke patients analyzed in the current study was somewhat lacking in statistical power, and large sample size studies are needed for further demonstration. Second, it was reported that corticospinal tract lesion load was a significant predictor of motor deficit (Zhu et al., 2010; Feng et al., 2015). However, in the current study, we focused on investigating whether subcortical stroke patients would exhibit abnormal dynamic characteristics of brain activity relative to healthy controls and to investigate whether the altered dynamic regional indexes were associated with clinical behavior in stroke patients. Hence, here, we did not give much thought to the characteristics of diffusion-tensor imaging of the patients. Third, the correlation analyses cannot pass the FDR or Bonferroni correction. Larger sample size will be necessary to confirm the current results in the future studies. Fourth, physiological noise of cardiac and respiratory cycles was not monitored during the MRI scanning, which may influence brain activity alterations. It is possible that alterations in network dynamics may reflect changes in brain state, since few constraints were imposed on a participant’s cognitive processes during the scanning. Finally, although the correlation analyses between dynamic regional indexes in regions showing significant group differences and size of lesion volume revealed no significant correlation, the heterogeneous clinical characteristics, such as lesion location, stroke severity and size of lesion volume, exhibited a relatively large variation across subjects and should be taken into consideration when interpreting the results.

## Conclusion

The pattern of intrinsic brain activity variability in multiple brain networks is altered in stroke patients with motor deficits compared with healthy controls. The alterations of dynamic brain activity in the SMN and DMN were correlated with the degree of motor functional impairment. Resting-state fMRI dynamic regional indexes might be potential tools to assess stroke patients’ motor function. Future studies will be needed to clarify the underlying mechanisms of alterations in the dynamic regional metrics after stroke.

## Author Contributions

JC contributed to the experiments, data analysis and writing of the manuscript. DS contributed to performing the experiments, and writing and revising the manuscript. YS contributed to the data collection. WJ designed the experiments and revised the manuscript. YW contributed to the data analysis and manuscript revision. QX contributed to the manuscript revision. CR is the guarantors of this study and had complete access to all data in the study. All authors are accountable for the contents of this research.

## Conflict of Interest Statement

The authors declare that the research was conducted in the absence of any commercial or financial relationships that could be construed as a potential conflict of interest.

## Footnotes

1 http://www.mccauslandcenter.sc.edu/mricro/mricron/

2 http://rfmri.org/DPARSF/
